# Clear aligner therapy practices among orthodontists practicing in Canada

**DOI:** 10.1186/s40510-024-00525-3

**Published:** 2024-07-08

**Authors:** Djessyca Miranda e Paulo, Letícia Fernanda Moreira-Santos, Maisa Costa Tavares, Tony Weir, Maurice J. Meade, Carlos Flores-Mir

**Affiliations:** 1 School of Dentistry, Federal University of Uberlândia, Uberlândia, Brazil; 2 Department of Pediatric Dentistry, School of Dentistry, Federal University of Minas Gerais, Belo Horizonte, Brazil; 3 Orthodontic Unit, Adelaide Dental School, Adelaide Health and Medical Sciences Building, The University of Adelaide, Adelaide, Australia; 4 Department of Dentistry, College of Health Sciences, Faculty of Medicine and Dentistry, University of Alberta, Edmonton Clinic Health Academy, Edmonton, AB 5-528, T6G 1C2 Canada

**Keywords:** Clear Aligner Appliances, Dental Health surveys, Orthodontics

## Abstract

**Background:**

The acceptability and preference for clear aligner therapy (CAT) has been increasing among orthodontists, but there is still a lack of consensus regarding CAT best practices. Consequently, this study aimed to investigate CAT practices among orthodontists practicing in Canada.

**Methods:**

The survey was conducted among orthodontists practicing in Canada using a modified previously published survey. Sixty orthodontists participated (6.1% response rate). It consisted of 11 sections with open and closed questions related to demographic information and particularities about using or not using CAT. The survey responses were exported from REDCap to a Microsoft Excel (Microsoft, Redmond, Wash) spreadsheet, then statistically analyzed using SPSS software (SPSS for Windows, version 21.0; IBM Inc., Armonk, NY, USA). The comments were categorized under themes and subthemes. Data were organized in descriptive statistics, expressing frequencies and percentages.

**Results:**

Almost 30% of the orthodontist’s annual caseload was treated with CAT, most frequently prescribed to adult patients. Case complexity and patient cooperation were the factors that most influenced the decision to prescribe CAT. Almost half of orthodontists reported sometimes combining CAT with adjunctive fixed appliances.

**Conclusions:**

Most orthodontists prescribe CAT, and its use is based on the malocclusion’s complexity. Orthodontists who do not prescribe CAT believe that fixed appliance therapy has superior treatment outcomes.

## Background

Clear Aligner Therapy (CAT) was popularized after the introduction of Invisalign® (Align Technology, Santa Clara, Calif.) [1]. Its growth was strongly supported by its aesthetic appearance and patient comfort, which directly impacts patients’ Oral Health-Related Quality of Life (OHRQoL) [2]. Its association with cutting-edge technology, such as CAD (computer-aided design) and CAM (computer-aided manufacturing), and extensive investment in merchandising further increased its popularity [3].

However, this technology has some limitations, mainly in treating complex malocclusions [1, 4]. So far, treatment success is reported to be mostly achieved in mild and moderate crowding cases [1, 4]. Frequently reported difficulties with CAT include patient adherence, finishing/detailing performance, closure of extraction spaces, specific tooth movements such as torque control, tooth rotations in rounded teeth, management of upper lateral incisors due to their size and morphology, and intrusion and extrusion movements [1, 5, 6].

This study aims to investigate CAT practices among orthodontists practicing in Canada, but more importantly, analyze the reasons for their use or not, as well as the malocclusion traits most frequently managed with CAT in this sample. Previous studies in the United States/Canada, United Kingdom, France, Australia, Republic of Ireland and New Zealand have reported increased acceptability and preference for CAT [1, 6–11]. However, orthodontists still lack consensus regarding the CAT best practices [1, 11].

## Materials and methods

The University of Alberta Research Ethics Board approved this study (ID Pro00128404). The survey was conducted among orthodontists practicing in Canada using a modified previously published survey (adapted from Meade & Weir, 2022) [9]. It consisted of 11 sections with open and closed questions related to demographic information and particularities about using or not using CAT. Section A addressed demographic characteristics. Section B assessed if respondents incorporated CAT in practice and the frequency of their use. If respondents answered that they did not use CAT, they were directed to Section K. Section C included questions regarding specific CAT choices and the reasoning behind them. Section D addressed digital treatment planning (DTP) protocols. Sections E and F included questions regarding case selection and treatment protocols. Section G contained questions about interproximal enamel reduction (IPR). Section H sought information about refinements and finishing. Section I aimed to determine the frequency of patient-reported CAT-related issues reported by orthodontists. Section J addressed aspects of CAT compared to fixed appliances. Section K addressed the factors influencing the decision not to provide CAT. The electronic questionnaire was adapted to the REDCap (Research Electronic Data Capture) software platform hosted at the University of Alberta. Data were collected and managed using REDCap tools. A pilot test was conducted among local community orthodontic colleagues to check survey validity, reliability, and acceptability. After modifications based on this feedback, the survey would take approximately 15 min to be completed.

The respondents were all active orthodontists (retired and student members were not considered) registered with the Canadian Association of Orthodontists (CAO), which comprised 981 orthodontic specialists at the survey date. The CAO was contacted in April 2023 to send the survey link to all active Canadian Association of Orthodontists members through their monthly bulletin. The provincial boards and local boards for orthodontists were also contacted individually by email, and they forwarded the survey link to its affiliates through email. After two weeks, a reminder email was sent to the provincial boards. The survey was closed in October 2023.

The survey responses were exported from REDCap to a Microsoft Excel (Microsoft, Redmond, Wash) spreadsheet, then statistically analyzed using SPSS software (SPSS for Windows, version 21.0; IBM Inc., Armonk, NY, USA). The comments were categorized under themes and subthemes. Data were organized in descriptive statistics, expressing frequencies and percentages.

## Results

Eighty-one active orthodontists accessed the email containing the link to the questionnaire. Among them, 60 agreed to participate in the study (overall response rate = 6.1%). Not all respondents answered all questions. Most responders were male (71.7%, *n* = 43) and obtained their specialist orthodontic qualification in Canada (60.0%, *n* = 36). A total of 95% (*n* = 57) of the respondents reported using CAT in clinical practice. Among them, the mean number of years in orthodontic practice was 19.0 (± 11.8), and 96.5% (*n* = 55) exclusively practiced in a private clinical setting.

Respondents reported that, on average, 29.9% (± 24.1%) of their annual caseload were treated with CAT. Table 1 indicates that most responders managed more than 50 CAT per year. Table 2 outlines that the complexity of the case influenced the decision of most responders to prescribe CAT. Table 3 shows that CAT was most frequently prescribed to adult patients. Most responders utilized more than one CAT brand, with Invisalign® being the most used (77.2%, *n* = 44), followed by 3 M Clarity® (3 M, St. Paul, Minn) at 10.5% (*n* = 6).

**Table 1:** Percentage of clear aligner therapy per year per orthodontist (*n* = 57)

Number of CATs per year	Orthodontist, % (*n*)
1–10	10.5 (6)
11–20	08.8 (5)
21–50	14.0 (8)
51–100	28.1 (16)
101–200	24.6 (14)
≥ 201	14.0 (8)

Note: CAT, clear aligner therapy

**Table 2:** Factors that influence decisions in prescribing clear aligner therapy (*n* = 57)

	Degree of influence % (*n*)			
Factors	No influence	Minor influence	Moderate influence	Major influence
Cost	41.1 (23)	33.1 (18)	19.6 (11)	7.1 (4)
Case complexity	5.4 (3)	5.4 (3)	21.4 (12)	67.9 (38)
Predicted patient cooperation	1.8 (1)	10.7 (6)	28.6 (16)	58.9 (33)
Patient demands	3.5 (2)	14.0 (8)	54.3 (31)	28.2 (16)
Patient expectations	5.4 (3)	12.5 (7)	57.1 (32)	25.0 (14)

**Table 3:** Patient types treated by orthodontists (*n* = 57)

	Frequency % (*n*)				
Patient type	Always	Mostly	Sometimes	Rarely	Never
Patients in the mixed dentition	0.0 (0)	7.1 (4)	19.6 (11)	37.5 (21)	37.5 (21)
Adolescents/teenage patients	1.8 (1)	15.8 (9)	66.6 (38)	12.3 (7)	3.5 (2)
Adult patients	3.6 (2)	62.5 (35)	33.9 (19)	1.8 (1)	0.0 (0)

The ease and quality of the DTP significantly influenced the choice of a particular aligner company/provider (Table 4). Only three (5.0%, *n* = 60) responders reported not changing the initially proposed DTP before acceptance. One to three sets of changes were made by 67.9% (*n* = 38), four to six sets of changes by 25.0% (*n* = 14), and more than six sets of changes were made by 1.8% (*n* = 1) before the digital plan was accepted. Table 5 shows that final movement protocols and tooth positions were the factors that mainly required changes from the initial DTP received from the CAT provider.

**Table 4:** Factors that influence decisions in choosing to use a particular aligner company/provider (*n* = 57)

	Degree of influence, % (*n*)			
Factors	No influence	Minor influence	Moderate influence	Major influence
Cost	10.9 (6)	29.1 (16)	38.2 (21)	21.8 (12)
Ease of digital treatment planning	1.8 (1)	5.5 (3)	30.9 (17)	61.8 (34)
Quality of digital treatment plan	1.9 (1)	5.6 (3)	31.5(17)	61.1 (33)
Sophistication of appliance features	1.8 (1)	10.9 (6)	41.8 (23)	45.5 (25)
Aesthetics of appliances	7.3 (4)	38.2 (21)	38.2 (21)	16.4 (9)
Patient satisfaction	7.3 (4)	16.4 (9)	47.3 (26)	29.1 (16)
Brand awareness among patients	12.7 (7)	40.0 (22)	20.0 (11)	27.3 (15)
Assistance in troubleshooting	9.1 (5)	30.9 (17)	34.5 (19)	25.5 (14)
Corporate support - advertising	25.5 (14)	47.3 (26)	20.0 (11)	7.3 (4)

**Table 5:** Factors in most need of a change from the initial plan received from the aligner provider (*n* = 57)

	Frequency % (*n*)				
Factor	Always	Mostly	Sometimes	Rarely	Never
Attachments	22.6 (16)	31.5 (17)	31.5 (17)	7.4 (4)	0.0 (0)
Movement protocols final	16.4 (9)	54.5 (30)	27.3 (15)	1.8 (1)	0.0 (0)
Tooth positions	35.2 (19)	35.2 (19)	22.2 (12)	7.4 (4)	0.0 (0)
Other	55.6 (5)	22.2 (2)	22.2 (2)	0.0 (0)	0.0 (0)

Regarding malocclusion traits, most responders reported feeling more comfortable treating spaced dentitions and mild crowding with CAT (Table 6). Table 7 shows that most respondents rarely or never used CAT in cases that required premolar extraction. Almost half (47.4%, *n* = 27) of orthodontists reported that they sometimes combined CAT with adjunctive fixed appliances, 40.4% (*n* = 23) rarely, and 8.8% (*n* = 5) never in the initial DTP.

**Table 6:** Comfort with treating different patient types with clear aligner therapy (*n* = 57)

Case type	Comfortable % (*n*)	Uncomfortable % (*n*)
Spaced dentition	100 (57)	0 (0)
Mild crowding (0–4 mm crowding)	100 (57)	0 (0)
Moderate crowding (4.1–8 mm crowding)	73.7 (42)	26.3 (15)
Severe crowding (> 8 mm crowding)	36.8 (21)	63.2 (36)
Deep overbite	66.7 (38)	33.3 (19)
Reduced overbite	89.5 (51)	10.5 (6)
Increased overjet	73.7 (42)	22.3 (15)
Reduced overjet	68.4 (39)	31.6 (18)
Posterior crossbite (unilateral)	47.4 (27)	52.6 (30)
Posterior crossbite (bilateral)	33.3 (19)	66.7 (38)

**Table 7:** Treatment modalities used with CAT (*n* = 57)

	Frequency % (*n*)				
Modality	Always	Mostly	Sometimes	Rarely	Never
Premolar extraction cases	0 (0)	3.5 (2)	17.5 (10)	35.1 (20)	43.9 (25)
Mandibular incisor extraction cases	17.5 (10)	17.5 (10)	47.4 (27)	10.5 (6)	7.0 (4)
Combined orthodontic/orthognathic surgery cases	3.5 (2)	7.0 (4)	28.1 (16)	19.3 (11)	42.1 (24)
Deep bite case with bite ramps	7.1 (4)	30.4 (17)	39.3 (22)	14.3 (8)	8.9 (5)
Cases combined with temporary anchorage devices	1.8 (1)	1.8 (1)	18.2 (10)	30.9 (17)	47.3 (26)

**Table 8:** Frequency of aligner change in different scenarios (*n* = 57)

	Frequency % (*n*)			
Scenario	Weekly	Fortnightly	Other	Not applicable
Mixed dentition	47.3 (26)	3.6 (2)	7.3 (4)	41.8 (23)
Adolescent/teenage	69.6 (39)	10.7 (6)	16.1 (9)	3.6 (2)
Adult patients	69.1 (38)	12.7 (7)	18.2 (10)	0 (0)

Most responders (71.9%, *n* = 41) did not routinely use Dental Monitoring® (Dental Mind, Paris, France) or other remote monitoring systems compared with 3.5% (*n* = 2) who always do. Table 8 shows that weekly aligner changes are preferred. The frequency of progress checks of CAT by responders was mainly performed at eight weeks (35.1%, *n* = 20) and 12 weeks (35.1%, *n* = 20) (Table 9).

Half of the responders (*n* = 28) mainly prescribed IPR to address the relief of crowding or reduction of potential dentoalveolar expansion in the initial digital plan (Table 10). Table 11 shows that 50.9% (*n* = 29) of responders reported that the amount of IPR provided was routinely less than the initial plan. The tools orthodontists considered always or mainly to carry out IPR were the perforated strips and the solid strip tools (Table 12).

**Table 9:** Frequency of clear aligner therapy progress checks by an orthodontist (*n* = 57)

Frequency	% (*n*)
Every 4 weeks	1.8 (1)
Every 6 weeks	5.3 (3)
Every 8 weeks	35.1 (20)
Every 12 weeks	35.1 (20)
Only if there is a problem—otherwise, give the patient all the aligners and see them at the end of treatment	3.5 (2)
When DM or remote monitoring system advises	1.8 (1)
Other	17.5 (10)

**Table 10:** Frequency of the prescription of interproximal reduction to address treatment objectives (*n* = 57)

	Frequency % (*n*)				
Treatment objective	Always	Mostly	Sometimes	Rarely	Never
Relief of crowding / Reduction of potential expansion	8.9 (5)	50.0 (28)	28.6 (16)	10.7 (6)	1.8 (1)
Reduction of black triangles	21.1 (12)	47.4 (27)	28.1 (16)	1.8 (1)	1.8 (1)
Adjustment of midlines	3.5 (2)	14.0 (8)	38.6 (22)	28.1 (16)	15.8 (9)
Adjustment of tooth-size discrepancies	23.6 (13)	40.0 (22)	32.7 (18)	3.6 (2)	0 (0)
Correction of overjet	0 (0)	14.0 (8)	47.4 (27)	24.6 (14)	14.0 (8)
Other	40.0 (2)	0 (0)	20.0 (1)	20.0 (1)	20.0 (1)

**Table 11:** Frequency of use of tools to perform interproximal reduction (*n* = 57)

IPR/additional plan	Frequency % (*n*)
More than the initial plans	12.3 (7)
Less than the initial plans	50.9 (29)
Both more and less than the initial plans	31.6 (18)
N/A. I do not have IPR prescribed in the additional aligners/refinement	5.3 (3)

Note: N/A, not applicable; IPR, interproximal reduction

**Table 12:** Tools used to carry out interproximal reduction

	Frequency, % (*n*)				
Tool	Always	Mostly	Sometimes	Rarely	Never
Perforated strip	28.8 (15)	28.8 (15)	19.2 (10)	15.4 (8)	7.7 (4)
Solid strip	19.6 (10)	13.7 (7)	29.4 (15)	17.6 (9)	19.6 (10)
Perforated disc	12.2 (6)	16.3 (8)	18.4 (9)	24.5 (12)	28.6 (14)
Solid disc	10.6 (5)	10.6 (5)	23.4 (11)	25.5 (12)	29.8 (14)
High-speed bur	4.3 (2)	10.6 (5)	19.1 (9)	21.3 (10)	44.7 (21)
Oscillating disc	2.3 (1)	4.5 (2)	6.8 (3)	11.4 (5)	75.0 (33)
Reciprocating strip	12.2 (6)	8.2 (4)	12.2 (6)	2.0 (1)	65.3 (32)
Other	16.7 (1)	0 (0)	0 (0)	0 (0)	83.3 (5)

The mean percentage of the responder’s annual CAT caseloads requiring one or more refinements was 97.1% (± 4.5). The responders reported a mean of 2.2 (± 1.1) refinements per patient. At the same time, the mean percentage of cases that begin with CAT and are completed with fixed appliances was 5.9% (± 13.1).

Table 13 shows patient-reported CAT-related issues by responders. None of the individuals noted problems occurred with a meaningful frequency. Regarding responders’ opinions on CAT, 20.0% (*n* = 11) disagreed, and 25.5% (*n* = 14) strongly disagreed that CAT provides superior treatment outcomes than fixed appliance therapy (Table 14). According to respondents who did not use CAT (*n* = 3), they decided not to provide CAT because they believe fixed appliance therapy provides better treatment outcomes.

**Table 13:** Patient-reported clear aligner therapy-related issues by an orthodontist (*n* = 57)

	Frequency % (*n*)				
Issue	Always	Mostly	Sometimes	Rarely	Never
Discomfort	1.8 (1)	3.6 (2)	38.2 (21)	54.5 (30)	1.8 (1)
Speech/lisping	1.8 (1)	12.5 (7)	39.3 (22)	42.9 (24)	3.6 (2)
Difficulty in compliance with wear protocol	3.6 (2)	18.2 (10)	67.3 (37)	10.9 (6)	0 (0)
Wear/breakage of aligner	0 (0)	0 (0)	39.3 (22)	53.6 (30)	7.1 (4)
Loss of aligner(s)	1.8 (1)	3.6 (2)	35.7 (20)	58.9 (33)	0 (0)
Poor fit of aligner(s)	1.8 (1)	1.8 (1)	50.0 (28)	44.6 (25)	1.8 (1)
Appearance of aligner(s)	0 (0)	3.6 (2)	12.7 (7)	50.9 (28)	32.7 (18)
Appearance/discomfort of attachments	0 (0)	16.1 (9)	39.3 (22)	39.3 (22)	5.4 (3)
Staining of teeth around attachments	0 (0)	16.1 (9)	44.6 (25)	33.9 (19)	5.4 (3)
Dissatisfaction with treatment length	0 (0)	7.1 (4)	44.6 (25)	44.6 (25)	3.6 (2)
Dissatisfaction with treatment outcome	0 (0)	3.7 (2)	20.4 (11)	68.5 (37)	7.4 (4)
Dissatisfaction with the need for refinement	0 (0)	7.1 (4)	32.1 (18)	50.0 (28)	10.7 (6)
Others	0 (0)	0 (0)	50.0 (1)	0 (0)	50.0 (1)

**Table 14:** Orthodontist opinions regarding clear aligner therapy duration and treatment quality (*n* = 57)

	Opinion, % (*n*)				
Statement	Strongly agree	Agree	Neither agree nor disagree	Disagree	Strongly disagree
CAT takes more time than FAT	16.1 (9)	7.1 (4)	39.3 (22)	19.6 (11)	17.9 (10)
CAT provides superior outcomes	3.6 (2)	7.3 (4)	43.6 (24)	20.0 (11)	25.5 (14)

Note: CAT, clear aligner therapy; FAT, fixed appliance therapy.

## Discussion

In the present study, we investigated CAT practices among orthodontists in Canada. Most respondents reported using CAT in their clinical practice, prescribing it in more than 50 cases annually. These findings reflect a trend due to CAT’s increased popularity and acceptability as an orthodontic treatment modality [1, 6–11]. Of approximately 981 orthodontists registered to CAO, 8% accessed the survey, but only 6% completed it. Though our survey experienced a low response rate, the response rate is like related surveys applied in other countries - with higher or lower response rates [1, 6–11].

A recent systematic review concluded that, despite low certainty, CAT might be associated with better OHRQoL than fixed appliances [2]. One of the main draws that make patients request CAT is their aesthetic characteristics [2, 11]. In this sample, patient preferences were a significant reported factor influencing decisions to prescribe CAT.

As reported in previous studies, adults comprised most of the patients undergoing CAT [9, 12, 13]. Lately, there has been an apparent rise in the number of teenagers being treated with CAT, potentially attributed to the CAT aesthetic appeal [1, 12, 13].

In our sample, the complexity of the case influenced the decision of most respondents to prescribe CAT, with cases requiring premolar extraction rarely or never being treated with CAT, as reported in previous studies in Australia, the United Kingdom, the Republic of Ireland, the USA, and New Zealand [1, 6–10]. Evidence supports that prescribing CAT is an alternative to fixed appliances in patients with mild-to-moderate malocclusion but not in more severe malocclusion cases [1, 4]. This is possibly due to the perception that CAT is less efficient than fixed appliances in torque control, resulting in less optimal occlusal contacts and post-treatment retention issues [5, 14]. Nearly half of the responders reported occasionally combining CAT with fixed appliances. This suggests that a treatment plan incorporating both approaches can optimize their respective advantages in managing certain malocclusions [6, 9, 15]. Orthodontists’ reports of the need to refine and complete CAT cases with fixed appliances may suggest a limitation in the DTP software to estimate individual tooth movements and treatment outcomes precisely [9, 16].

Jaber et al. suggested that CAT and fixed appliances are equally effective in orthodontic extraction cases [17]. However, treatment duration was shorter with fixed appliances than with CAT, as they offered the advantage of achieving better buccolingual inclination and occlusal contacts in a shorter treatment time [5, 17].

In the present study, most responders use more than one brand, with Invisalign® being the most common. Similar findings were previously found in the United States, the United Kingdom, Australia, and New Zealand [1, 6, 9]. Moreover, brand advertising weakly influences orthodontists’ decision-making regarding CAT prescriptions, whereas the ease and quality of DTP have a moderate or significant impact [3, 9]. Most respondents indicated that they made one to three modifications to the DTP before its approval. A recent survey by Meade et al. (2023) revealed a similar finding in patients undergoing non-extraction treatment with the Invisalign® appliance [6]. Our respondents predominantly cited adjustments from the initial plan related to final movement protocols and tooth positions.

In our sample, orthodontists do not routinely use remote monitoring systems. Current evidence contradicts remote monitoring’s capability to reduce treatment duration and the number of in-person visits [18, 19]. However, remote monitoring is a business decision since its current costs may be a limiting factor [18].

In this survey, most orthodontists reported adopting a weekly aligner change protocol. A randomized clinical trial result indicated that a 7-day protocol appeared effective, as there was no notable clinical distinction compared to protocols lasting 10 or 14 days [20]. Notably, employing a 7-day protocol did lead to a significant reduction in the overall treatment duration. The authors also suggested that, especially in instances necessitating intricate adjustments of posterior teeth, a 14-day interval for changing aligners could prove advantageous [20].

During CAT, IPR is a frequently used technique for creating spaces to align teeth and improve long-term alignment stability [21]. According to the respondents in our sample, the amount of IPR finally provided was expected to be less than initially planned − a pattern consistent with findings from previous investigations [9, 21–23]. Overall, these studies have concluded that the in vivo removal of interproximal enamel was generally less than the initially planned IPR. This finding raises the possibility of shortcomings in the DTP, deviations from the initial DTP specifications during IPR implementation, and potential inadequacies in the clear aligners software systems [9]. It has also been suggested that creating interproximal spaces are needed while attempting to move teeth with CAT; the argument is that those spaces improve the grip of the aligner material around the teeth, improving the overall tooth movement efficiency. No references have been identified to support or refute this hypothesis.

In the present study, the belief that fixed appliances provide better outcomes than CAT may have influenced responders’ decision not to prescribe CAT. Nearly half of orthodontists who prescribed CAT disagreed or strongly disagreed that it offered superior results to fixed appliances. Therefore, the finding that orthodontists in the sample believe that CAT does not yield superior treatment outcomes may be because it is indicated based on the case’s complexity, making a “subjective” comparison between CAT and fixed appliance cases challenging.

The limitations included the relatively low response rate and recall bias, which were still higher than in other studies. Another issue we faced was orthodontists’ compliance in answering the lengthy questionnaire, leading to incomplete responses. A saturation of online research in the field may also explain the lack of interest among respondents [24].

In addition, orthodontists who do not prescribe CAT may have declined participation, as the survey explicitly focused on CAT prescription, as indicated in the survey title. Consequently, individuals using CAT might have shown a higher inclination to participate, introducing recruitment bias and potentially leading to overestimating CAT prevalence among respondents.

## Conclusions

Within the limitations of this study, the assessment of CAT practices among orthodontists practicing in Canada showed that most of them prescribe CAT. CAT use is based on the malocclusion’s complexity. Furthermore, the ease and quality of the digital planning interface influence the option of a particular aligner company/provider, with one to three modifications to the DTP being more frequently reported before its approval. A mean of 2.2 refinements per patient was reported during treatment.

The amount of IPR performed was believed to be lower than initially planned by the DTP software. Orthodontists who do not prescribe CAT do not do so because they believe that fixed appliance therapy has superior treatment outcomes. The results in the present study broadly match those reported in several recent studies in Australia, the United Kingdom, the Republic of Ireland, the USA, and New Zealand, indicating a broadly similar landscape of CAT practice across all nations thus far surveyed.

## Data Availability

The datasets used and/or analyzed during the current study are available from the corresponding author upon reasonable request.

## Abbreviations

CAD: Computer-aided design
CAM: Computer-aided manufacturing
CAO: Canadian Association of Orthodontists
CAT: Clear aligner therapy
DTP: Digital treatment planning
IPR: Interproximal enamel reduction
OHRQoL: Oral health-related quality of life
REDCap: Research Electronic Data Capture
